# Discovery of PD-L1 Peptide Inhibitors from Ascidian Enzymatic Hydrolysates by Affinity Ultrafiltration Coupled to NanoLC-MS/MS

**DOI:** 10.3390/md23040137

**Published:** 2025-03-21

**Authors:** Qiuyang Huang, Xiaoling Zang, Xinyu Jin, Qian Liu, Xin Zhang, Xinyu Li, Lizhen Zhao, Zhihua Lv

**Affiliations:** 1 School of Medicine and Pharmacy, Ocean University of China, Qingdao 266003, China; 18410730350@163.com (Q.H.); 21220813115@stu.ouc.edu.cn (X.J.); liuq@stu.ouc.edu.cn (Q.L.); zhangxin199810@gmail.com (X.Z.); 21220811030@stu.ouc.edu.cn (X.L.); 2 Laboratory for Marine Drugs and Bioproducts, Qingdao Marine Science and Technology Center, Qingdao 266237, China; 3 Sinopep Allsino Bio Pharmaceutical Co., Ltd., Hangzhou 310020, China; 4 College of Physics, Qingdao University, Qingdao 266071, China; zhaolz@qdu.edu.cn

**Keywords:** *Ciona intestinalis*, *Styela clava*, PD-L1 peptide inhibitors, affinity ultrafiltration (AUF)-nanoLC-MS/MS, hydrolysates, trypsin

## Abstract

Anti-PD-1 and anti-PD-L1 antibodies have achieved great clinical success in cancer immunotherapy, and peptide and small molecule inhibitors of PD-1/PD-L1 binding also attract much attention. Ascidians are not only seafood, but are also an important source of bioactive substances, including anti-tumor components. In this study, ascidian enzymatic hydrolysates were found to contain PD-1/PD-L1 inhibitory components. Affinity ultrafiltration (AUF) coupled with the nanoLC-MS/MS method was first applied in screening for PD-L1 peptide inhibitors from ascidian enzymatic hydrolysates. Two anti-PD-L1 ascidian peptides, C5 (LDVVIHTVTYGDR) and S2 (VLRDNIQGITKPAIR), were filtered out from the ascidians *Ciona intestinalis* and *Styela clava*, respectively. C5 and S2 showed moderate anti-PD-1/PD-L1 effects with the IC_50_ values of 33.9 µM (C5) and 112.8 μM (S2), respectively, by homogenous time-resolved fluorescence (HTRF) binding assay, and the K_D_ values of 22.9 µM (C5) and 29.1 µM (S2), respectively, by surface plasmon resonance (SPR) assay. The results of this study suggest that ascidian enzymatic hydrolysates may be a potential source of bioactive peptides with anti-PD-1/PD-L1 activity.

## 1. Introduction

Marine organisms are a treasure trove rich in protein and peptide components [1]. Marine ascidians, or sea squirts, are farmed and fished in some parts of the world, e.g., Japan and Korea, and consumed as food due to its high nutritional and medicinal value [2,3], and also serve as food for some fishes [4]. Ascidians are filter-feeders, belong to urochordates and tunicates [5], and produce a high chemical diversity of secondary metabolites that are one of the richest sources of bioactive compounds. The secondary metabolites from ascidians and symbionts include alkaloids, peptides, and polyketides, which contribute to the development of new medicinal substances [6]. Alkaloids are the most prominent family of compounds that have anti-tumor activities [7,8]. The alkaloid Trabectedin (ET-743) [9,10] isolated from the ascidian *Ecteinascidia turbinate* was the first anti-tumor drug from ascidians [5,11].

Ascidian bioactive peptides have potential biological functions of antineoplastic, antiviral activity, antidiabetic, antioxidant activity, and immunomodulatory properties [12,13]. Bioactive peptides from ascidians have three types of structures, cyclic peptides, depsipeptides, and linear peptides [13]. The ascidian-derived peptides with possible cytotoxic potentials were reported [13], such as cyclodepsipeptide Plitidepsin (Aplidine) [14,15], Lurbinectedin (PM01183) [16], depsipeptide Didemnin B [17,18], thiazoline-containing peptide Trunkamide A [19,20], depsipeptides Tamandarin A and B [21], cyclic heptapeptides Mollamides [22,23], cyclic peptide Vitilevuamide [24,25], cyclic peptides Bistratamides M and N [26], iodobenzene-containing dipeptides [27], and cysteine-rich antimicrobial peptides [28]. Plitidepsin (Aplidine) was approved in Australia in 2018 for the treatment of multiple myeloma, leukemia, and lymphoma [14,29]. Lurbinectedin and Didemnin B were under clinical trials [13].

In recent years, anti-PD-1 and anti-PD-L1 antibodies have achieved great clinical success in cancer immunotherapy. In addition, peptide and small molecule inhibitors of PD-1/PD-L1 also attract much attention. Antitumor peptides have some advantages in comparison with antibodies, e.g., low production costs, easy to transport and store, and good stability [30]. Programmed death ligand 1 (PD-L1) is a transmembrane protein consisting of 290 amino acids [31], also known as B7 homolog 1 (B7-H1) or cluster of differentiation (CD274) [32]. PD-L1 together with PD-L2 (CD273) serve as ligands of programmed death 1 (PD-1). PD-L1 is normally expressed in various immune cells in the body, including T cells, B cells, dendritic cells, and macrophages [33], and PD-L1 is also upregulated in tumor cells to exhaust tumor infiltrating lymphocytes [34,35]. The equilibrium dissociation constant (K_D_) values of PD-1 with immobilized PD-L1 proteins were reported to be 1.56 μM, 1.15 μM, and 8.2 μM, respectively [36,37,38]. Nowadays, the PD-1 and PD-L1 inhibitors are research hotspots in cancer immunotherapy [39], and monoclonal antibody (mAb)-based immunotherapy has been considered as a main component of cancer therapy [35].

With continuous progress of modern separation and analysis techniques, high-throughput screening methods for enzyme inhibitory peptides have emerged as powerful tools in the discovery of bioactive peptides [32]. Recently, the methodologies based on the phage display library, bacteria display library, and peptide fragment of proteins have been applied in screening for PD-L1 inhibitors [36,40,41,42,43]. Using phage display technique, Chang et al. reported a D-form peptide ^D^PPA-1 (NYSKPTDRQYHF), which had a high affinity to PD-L1 with a K_D_ value of 0.51 μM measured by surface plasmon resonance (SPR), and ^D^PPA-1 at 2 mg/kg inhibited the tumor growth in CT26 tumor-bearing mice and prolonged their survival time to nearly 50% longer [41]. Li et al. used the bacterial display library to screen out a peptide (TPP-1) (SGQYASYHCWCWRDPGRSGGSK), which specifically bound to PD-L1 with a very high affinity, K_D_ = 0.09467 μM by SPR [42]. Zou et al. reported a peptide from human peroxiredoxin-5 (hPRDX5), with an IC_50_ of 0.646 μM [40].

The cyclic peptide inhibitors against PD-L1 were reported with high inhibitory activities [44,45,46,47]. The macrocyclic peptide pep-57 bound with PD-L1 (patented by Bristol-Myers Squibb Company) was tested by Ganesan et al. with a K_D_ value of 19.88 nM and an IC_50_ of 7.68 nM against the PD-1/PD-L1 binding [44]. Zhai et al. reported a cyclic peptide C8 (CKWYRPSEC) using phage display technique, with a K_D_ value of 0.64 μM by microscale thermophoresis (MST) method, and a PD-1/PD-L1 inhibitory IC_50_ < 10 μM [46].

Notably, a superior binding affinity to PD-L1 may result in significant toxicity to healthy cells with low-level PD-L1 expression [43]. Park et al. reported that the chimeric antigen receptor (CAR)-T cells with mM affinity to intercellular adhesion molecule-1 (ICAM-1) only attacked tumor cells with high levels of ICAM-1, and achieved more rapid tumor elimination and more safety than the CAR-T cells with nM affinity to ICAM-1 [48].

There have been no reports on the application of affinity ultrafiltration (AUF) methodology combined with nanoLC-MS/MS in screening for PD-1 or PD-L1 peptide inhibitors. AUF-nanoLC-MS/MS is expected to be a promising tool in high-throughput screening of peptide binders for a target protein. Using the AUF-nanoLC-MS/MS method, peptide binders to a protein are extracted based on the affinity of peptides to the target protein in a solution phase, and are identified using nanoLC-MS/MS. This method is easy to operate and highly efficient for extracting bioactive molecules from complex chemical substrates [49].

The studies of PD-L1 peptide inhibitors from marine organisms are scarce. To our knowledge, ascidian-derived peptide inhibitors of PD-L1 have not been reported yet. The Shandong peninsula of China has a unique geographical location and is rich in ascidian resources, such as the ascidians *Ciona intestinalis* and *Styela clava*. In this study, we aim to expect to screen potential PD-L1 inhibitory peptides with anti-tumor activity from *Ciona intestinalis* and *Styela clava* hydrolysates by AUF-nanoLC-MS/MS and enrich the research on anti-PD-1/PD-L1 marine peptides.

## 2. Results and Discussion

### 2.1. Anti-PD-1/PD-L1 Effects of Total Enzymatic Hydrolysates of Ascidians

In vitro homogenous time-resolved fluorescence (HTRF) assays were used to examine the inhibitory activity of total enzymatic hydrolysates of ascidians on PD-1/PD-L1 interaction. At a final concentration of 50 mg/mL, the inhibition ratios of *Ciona intestinalis* and *Styela clava* enzymatic hydrolysates against PD-1/PD-L1 binding were found to be 96.6% and 99.2%, respectively. The results indicated the presence of PD-1/PD-L1 inhibitory components in ascidian enzymatic hydrolysates, suggesting that ascidians have a potential immunotherapy function that is rarely found in other foods. These total ascidian hydrolysates may directly find an application as bioactive hydrolysates. However, it is necessary to conduct in-depth study on which components play a key bioactive role, and then these bioactive agents could be optimized and synthesized for potential medicinal use.

### 2.2. Filtration of PD-L1 Peptide Inhibitors Using AUF

Taking enzymatic hydrolysates of *Styela clava* as an analyte, the AUF method for filtration of PD-L1 peptide inhibitors was first optimized. The concentrations of 50 and 100 mg/mL of *Styela clava* enzymatic hydrolysates and usage or no usage of ultrasonic treatment during the dissociation of PD-L1 binders from PD-L1 proteins were tested. The 100 µL of 50 mg/mL enzymatic hydrolysates were incubated with 100 µL of 50 µg/mL PD-L1 for 60 min at 37 °C, then 200 µL of methanol/water (90:10, *v/v*) was added to a filter tube to separate the molecules bound to PD-L1 from PD-L1 proteins, with a 30 min sonication at room temperature. It was found that the PD-L1 peptide binders in the enzymatic hydrolysate could be effectively filtered out using the AUF method, having an 86.1% ± 0.1 inhibition (three analyses for a single hydrolysate sample) on PD-1/PD-L1 interaction by HTRF assay.

When the sample concentration of 50 mg/mL was increased to 100 mg/mL, the inhibition ratio of the AUF filtrates on the PD-1/PD-L1 binding decreased to 18.4% ± 0.3. One of the reasons for such an inhibition ratio decrease with an increased sample concentration is that a high concentration of enzymatic hydrolysates may hinder the binding of active agents towards PD-L1. Additionally, a high concentration of samples may be excessively adsorbed onto the ultrafilter membrane that obstructed the outflow of the filtered PD-L1 binders. Therefore, a 50 mg/mL concentration of enzymatic hydrolysates in this study was appropriate in screening for PD-L1 peptide binders.

In addition, it was noted that when only 200 µL of methanol/water (90:10, *v/v*) was added to a filter tube at the separation of the peptides bound to PD-L1 from PD-L1 proteins without sonication; no obvious PD-1/PD-L1 inhibitory components +were detected in the AUF filtrates, indicating the importance of sonication at the separation step of AUF. Together, in screening for PD-L1 peptide binders from ascidian enzymatic hydrolysates, we adopted a concentration of 50 mg/mL enzymatic hydrolysates and 30 min sonication for the separation of PD-L1-bound peptides from PD-L1.

### 2.3. Identification of PD-L1 Peptide Binders by nanoLC-MS/MS

**Figure 1:**
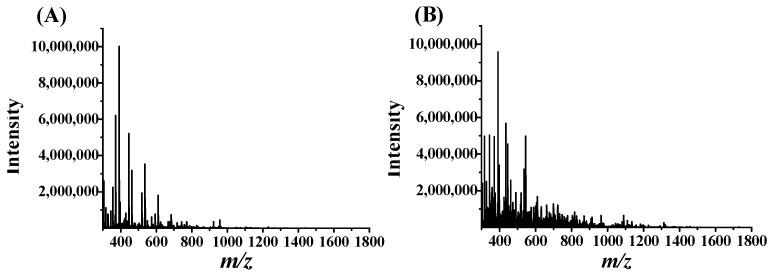
Total peak intensity chromatograms of the AUF filtrates from and *Ciona intestinalis* hydrolysates (**A**) and *Styela clava* hydrolysates (**B**), incubated with PD-L1.

PD-L1 peptide binding agents from ascidian enzymatic hydrolysates by the AUF extraction were identified using the nanoLC-MS/MS method. Figure 1 showed total peak intensities of the AUF-extracted PD-L1 binders from *Ciona intestinalis* and *Styela clava* enzymatic hydrolysates. An obvious difference was seen between the AUF filtrates from *Ciona intestinalis* and *Styela clava* enzymatic hydrolysates (Figure 1). The *b* series and *y* series ions detected by MS were searched using MaxQuant combined with the UniProt ascidiacea database to determine peptide sequences. The 42 and 24 peptides binding to PD-L1 by the AUF extraction were achieved from *Ciona intestinalis* and *Styela clava* enzymatic hydrolysates, respectively (Table 1, Table 2, Table 3 and Table 4). When comparing these peptides with those present in PD-L1 or trypsin, no identical sequences were found.

Peptide Ranker [50] was used to predict the properties of the peptides filtered out by AUF. HPEPDOCK [51] was used for blind peptide-protein docking with a hierarchical algorithm. For each peptide-protein docking, 100 docking poses were generated and ranked by docking scores. The lowest docking scores for each peptide were listed in Table 2 and Table 4, most of which showed good binding capability with PD-L1, which showed the advantage and specificity of the AUF method. Considering possible difficulty in synthesizing peptides with long sequences and the fact that natural bioactive peptides often consist of 2–20 amino acids [52], we preferentially selected the peptides with less than 22 amino acids for subsequent validation of anti-PD-L1 activity, and C1 and C4–C8 from *Ciona intestinalis* and S1–S3 from *Styela clava* were selected to be synthesized according to the HPEPDOCK docking results (Figure S1).

### 2.4. Anti-PD-1/PD-L1 Effects of PD-L1 Peptide Binders

**Figure 2:**
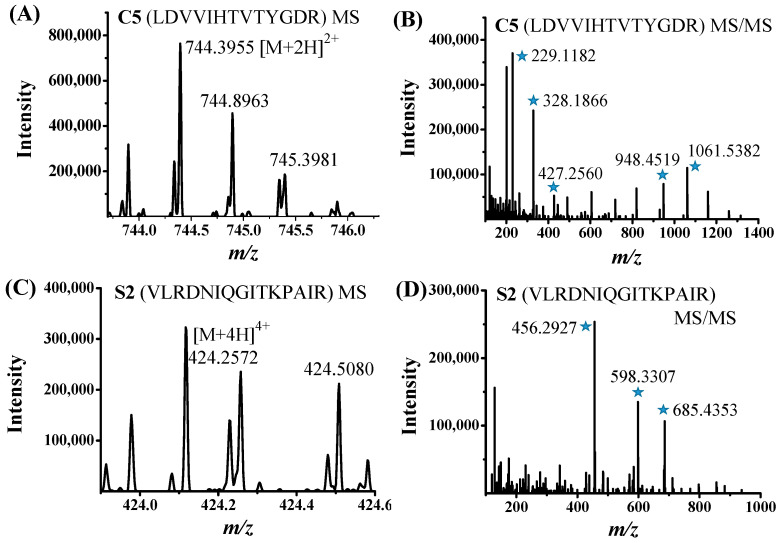
MS and MS/MS spectra of peptides C5 (**A**,**B**) and S2 (**C**,**D**). MS/MS fragment ions with *m/z* values that matched those of theoretical *b* and *y* fragment ions of the peptides were marked with asterisks.

**Figure 3:**
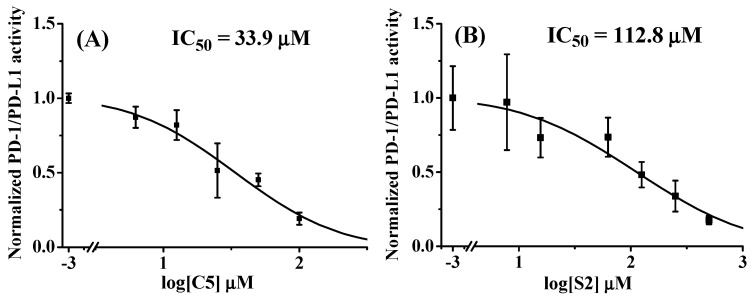
Inhibitory effects of C5 (**A**) and S2 (**B**) on PD-1/PD-L1 interaction with three repetitions for each concentration by the HTRF assays.

The PD-1/PD-L1 inhibition ratios of nine selected peptides (C1, C4–C8, and S1–S3) derived by the AUF-nanoLC-MS/MS method were tested by the HTRF assay with a maximal concentration of 50 μM. Among the nine peptides, C5 and S2 were found to exhibit largest inhibitory effects on PD-1/PD-L1 interaction. The MS and MS/MS spectra of C5 and S2 were displayed in Figure 2. C5 and S2 were confirmed to have concentration-dependent inhibition against PD-1/PD-L1 interaction. In the HTRF experiments, the IC_50_ values of C5 and S2 were fitted to be 33.9 µM and 112.8 µM, respectively (Figure 3). C5 and S2 have high percentage of branched amino acids residues, 38.5% and 33.3%, respectively. The unusual structures of marine-derived peptides, such as high percentage of proline and branched amino acids residues, cyclic and depsipeptide structures make the peptides less likely to be recognized by digestive enzymes, and, thus, may increase their in vivo stability [53].

**Figure 4:**
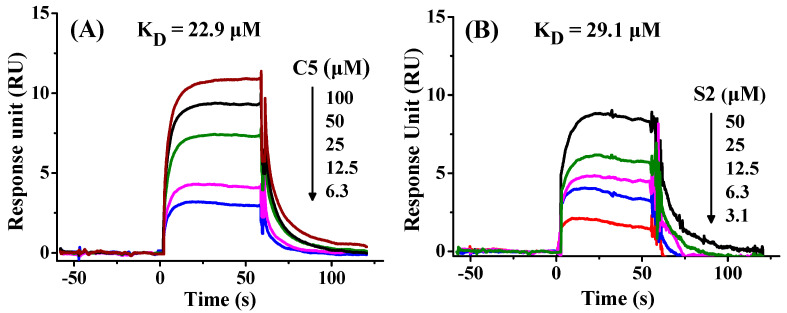
Binding affinities of C5 and S2 to PD-L1 by SPR. Equilibrium response versus concentration for C5 (**A**) and S2 (**B**). K_D_ = k_off_/k_on_, where k_on_ and k_off_ are association and dissociation rate constants, respectively. Line colorcode: brown: 100 μM; black: 50 μM; green: 25 μM; magenta: 12.5 μM; blue: 6.3 μM; red: 3.1 μM.

The binding affinity of C5 and S2 to PD-L1 was evaluated by SPR. The recombinant human PD-L1 (residue 18–239, extracellular domain) was immobilized on a CM5 sensor chip by amino coupling. The K_D_ values of C5 and S2 were 22.9 µM and 29.1 µM, respectively (Figure 4A,B), which were comparable to the K_D_ value of the positive control BMS202 (16.7 μM) [54]. The K_D_ values of C5 and S2 confirmed their binding ability with PD-L1, though their K_D_ values were much larger than the reported K_D_ values of macrocyclic peptide BMS pep-57 (19.88 nM) [44], D-form peptide ^D^PPA-1 (0.51 μM) from phage display library [41], TPP-1 (0.09467 μM) from bacterial display library [42], CLP002 (0.366 μM) [43], cyclic peptide C8 (0.64 μM) [46], and cyclic peptide 66 (5.67 μM) [47]. From another perspective, the very large binding affinity of the molecules bound to PD-L1 may also have large toxicity to healthy cells with low expression levels of PD-L1 [43]. The PD-L1 peptide inhibitors from ascidians could possess less toxicity compared to peptide inhibitors with very small K_D_ values.

The amino acid sequence of C5 is LDVVIHTVTYGDR. Upon searching in UniProt, uncharacterized proteins of *Streptomyces albireticuli* and *Streptomyces* sp. *ISL-11* contain a sequence fragment LVVVHTVTYGQR, and *Streptomyces tubercidicus* has a sequence fragment LVVVHTVTYGKR, which is similar to that of C5 derived in this study. Ascidians can produce a variety of bioactive secondary metabolites, and many of these active products are not produced by ascidians themselves, but by their symbionts [5]. *Streptomyces* is the most diverse actinomycete that widely exists in many ascidians [55,56,57]. It may be suggested that C5 might come from *Streptomyces* on ascidians, which needs to be further evaluated. Ascidians, especially parts of their inner body tissues, are used for flavoring foods for human consumption, and ascidian body may provide anti-PD-1/PD-L1 immunotherapy components. The sequence of S2 (VLRDNIQGITKPAIR) exactly matches that of Histone H4 of *Ciona savignyi*. Histone H4 is the core component of nucleosomes, which wrap and compact DNA into chromatin.

### 2.5. Predicted Binding Sites of C5 and S2 Towards PD-L1

**Figure 5:**
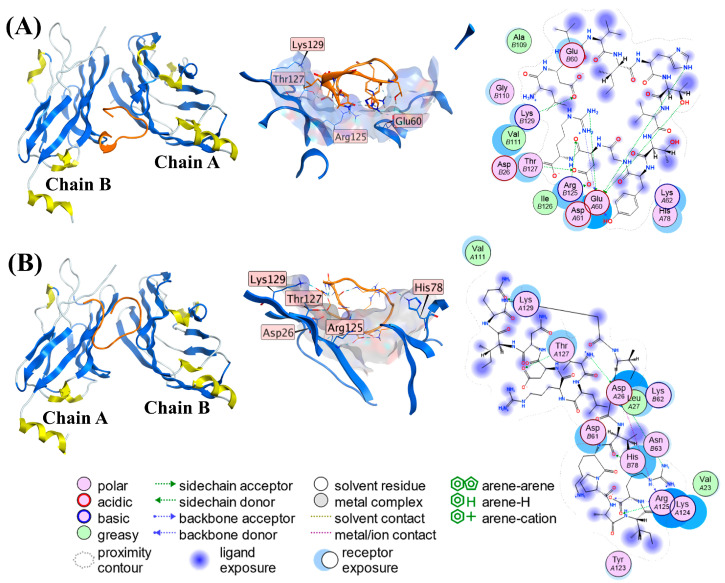
Three-dimensional and 2D representations of interactions between C5 (**A**) or S2 (**B**) and PD-L1 using molecular docking by MOE. The loops in the orange color in the 3D images represent C5 or S2.

The MOE docking scores of C5 and S2 with PD-L1 were -42.99 and -45.35 kcal/mol, respectively. As shown in Figure 5, C5 and S2 formed 10 (C5) and 8 (S2) hydrogen bonds, 4 (C5) and 8 (S2) ionic interactions, respectively (Table 5). At the same time, both C5 and S2 interacted with the Thr127 residue via hydrogen bond. Although the interface between C5 or S2 and PD-L1 were not hot regions involved in the PD-1 and PD-L1 binding [58], they induced spatial steric hindrance and conformational changes, reducing PD-1/PD-L1 interaction. C5 formed both a H-bond and an electrostatic interaction with Lys129, three H-bonds and two ionic interactions with Arg125, five H-bonds, and an ionic interaction with Glu60, and a H-bond with Thr127. S3 formed two H-bonds and four ionic interactions with Asp26, a H-bond with Lys129, Thr127, and His78, respectively, and three H-bonds and four ionic interactions with Arg125 (Table 5).

In view of the anti-PD-1/PD-L1 IC_50_s, K_D_ values, and docking sites, C5 and S2 were shown to have an ability binding to PD-L1, and hydrogen bonds and ionic interactions were formed between C5 or S2 and PD-L1. Ascidians can be expected to be a potential natural source of anti-PD-1/PD-L1 peptides, in addition to being a nutritional sea food.

## 3. Materials and Methods

### 3.1. Preparation of Trypsin Hydrolysates of Ascidians

Fresh *Ciona intestinalis* and *Styela clava* were collected from Weihai, Shandong of China. The 250 mL of 0.2 mol/L potassium dihydrogen phosphate solution and 118 mL of 0.2 mol/L sodium hydroxide solution were diluted with water to 1000 mL to obtain a mixture solution. Whole *Ciona intestinalis* and whole *Styela clava* were washed with tap water and were lyophilized and pulverized into powders using a grinder. The freeze-dried crude powders of *Ciona intestinalis* and *Styela clava* were added to a mixture solution a ratio of 1 g/20 mL and then placed in a water bath at 50 °C. The pH was adjusted to 6.8. Then, trypsin (Solarbio, Beijing, China) was added at a ratio of enzyme to substrate of 8000 U/g, and the enzymolysis time was 8 h. Finally, after denaturation of trypsin with boiling water at 100 °C for 15 min and centrifuged at 6000 rpm/min for 20 min, the supernatants were lyophilized and stored at −80 °C.

The protein content was determined using a bicinchoninic acid (BCA) assay kit (Beyotime, Shanghai, China). Briefly, 20 μL of sample was mixed with BCA working reagent prepared by mixing 50 volumes of BCA reagent A with 1 volume of BCA reagent B (50:1). The plate was incubated for 30 min at 37 °C. Absorbance at 562 nm was measured (Multiskan FC, Thermo scientific).

### 3.2. AUF Experiment

**Figure 6:**
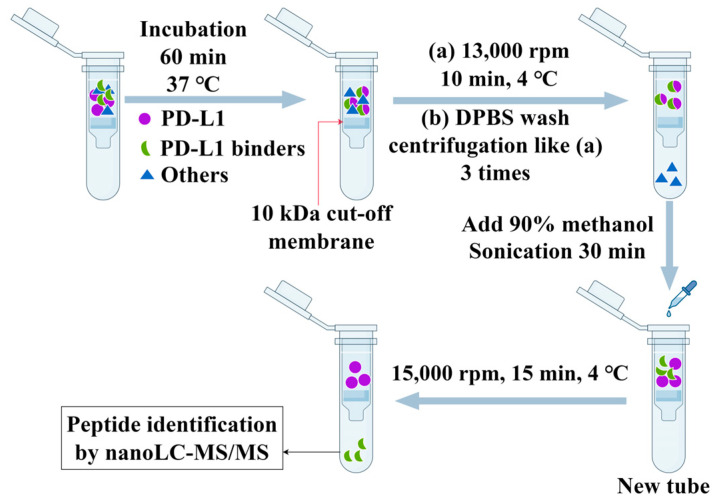
Flowchart of the AUF experiment to screen for PD-L1 peptide binders from ascidian enzymatic hydrolysates.

The peptide binders of PD-L1 were screened out through incubation, washing, and elution steps in the AUF experiment (Figure 6). A volume of 100 μL enzymatic hydrolysates at 50 mg/mL was incubated with 100 μL of 50 µg/mL PD-L1 (25 kDa, Abcam, Cambridge, UK) and incubated at 37 °C for 90 min under shaking. Next, the filtration was conducted using a 10 kDa cut-off membrane, and the sample was centrifuged at 13,000 rpm for 10 min at 4 °C and washed by the addition of 200 μL of DPBS, repeated 3 times to remove the unbound compounds of PD-L1. Subsequently, the binders were released from PD-L1 proteins by addition of 200 μL of 90% methanol with sonication for 30 min. Then, the sample with the peptide binders was centrifuged at 15,000 rpm/min for 15 min at 4 °C, and the filtrates were dried under reduced pressure for subsequent nanoLC-MS/MS analysis.

### 3.3. NanoLC-MS/MS Analysis

The nanoLC-MS/MS analysis was performed on a system composed of an EASY-nLC 1200 (Thermo Fisher Scientific, Inc., Waltham, MA USA) that was interfaced via a nanospray flex ion source to a Q Exactive orbitrap mass spectrometer (Thermo Fisher Scientific, Inc.). The nLC was operated with a Waters nanoEase M/Z Peptide BEH C18 analytical column (75 μm × 150 mm, 1.7 μm particles with 300 Å pores) at room temperature. The spray voltage and capillary temperature were set as 2.3 kV and 300 °C, respectively. Each sample redissolved in ddH_2_O was injected to nLC with a volume of 2 μL, and a 60 min LC gradient of solvent A (0.1% formic acid (*v/v*)) and solvent B (80% acetonitrile, 0.1% formic acid (*v/v*)) at a flow rate of 350 nL/min. The LC gradient was 6% B in 0–6 min, 25% B in 6–25 min, 45% B in 25–45 min, 100% B in 45–55 min, and 100% B in 55–60 min. All LC-MS/MS data were collected using XCalibur (Thermo Fisher Scientific). The survey scans (*m/z* 300–1800) (MS1) were acquired in the orbitrap at a resolution of 70000, and the 10 most abundant precursors in each survey scan were analyzed using the HCD-MS/MS scans at a resolution of 17,500, i.e., one survey scan (MS1) and 10 dependent MS/MS scans.

MS/MS data were searched using MaxQuant software version 2.4.11.0 combined with UniProt (https://www.uniprot.org/, accessed on 1 June 2022) ascidiacea database. The following parameters were used: false discovery rate (FDR) < 0.01 for peptide identification, a mass tolerance of 20 ppm for both MS1 precursor and MS/MS spectra, trypsin/P as proteolytic enzyme, up to 2 missed cleavages for trypsin, carbamidomethylation of cysteine as fixed modification, and oxidation of methionine and N-terminal acetylation as variable modifications. For an identified peptide, at least one unique peptide fragment should be identified.

### 3.4. Molecular Docking Using HPEPDOCK and MOE

HPEPDOCK web server [51] was used for peptide-protein docking. The crystal structure of PD-L1 (PDB code: 5J89) from the PDB protein database was used [59]. The import files of HPEPDOCK included the PD-L1 PDB file and the peptide sequence in FASTA format. The number of conformations was set to 1000, and the number of output peptide binding structures for each docking was set to 100. The docking structures with the best docking scores were finally selected.

In addition, the C5 and S2 dockings with PD-L1 were calculated by the molecular operating environment (MOE) to predict the interaction between C5 or S2 and PD-L1, after the PD-L1 protein structure was repaired and hydrogenated. The peptide binding site was searched by site finder and the binding site with the lowest docking score was chosen. In the peptide docking with PD-L1 using MOE, the Amber10 force field and Generalized-Born Volume Integral/Weighted Surface Area (GBVI/WSA) dG scoring were calculated.

### 3.5. HTRF Binding Assay

The candidate anti-PD-L1 peptides were synthesized by GuoPing Pharmaceutical Co., Ltd. (Anhui, China), and the MS diagrams of the synthesized peptides were shown in Figure S1. The inhibitory effects of the peptides on PD-1/PD-L1 interaction were evaluated using the PD-1/PD-L1 HTRF binding assay kit from Cisbio company (Shanghai, China) according to the manufacturer’s instructions. Briefly, 2 µL analyte solution, 4 µL Tag1-PD-L1 protein, and 4 µL Tag2-PD-1 protein were incubated together for 15 min at room temperature. Next, 10 µL of pre-mixed anti-Tag1-Eu3^+^ (HTRF donor) and anti-Tag2-XL665 (HTRF acceptor) were added. The plate was sealed and incubated for 2 h at room temperature. Then, the plate sealer was removed and the plate was read by a Tecan Spark^TM^ multimode microplate reader, and the fluorescence resonance energy transfer (FRET) ratio values (HTRF ratios) were measured as a ratio of 665 nm/620 nm fluorescence ×10,000. Normalized PD-1/PD-L1 activity = average (HTRF ratio of the analyte—HTRF ratio of the blank)/average (HTRF ratio of 0 µM analyte—HTRF ratio of the blank) ± standard deviation. Three replicates were carried out for each analyte concentration. Three independent experiment repetitions were carried out. IC_50_ was calculated by SPSS version 25.

### 3.6. SPR Experiment

The SPR experiment was performed on a Biacore T200 instrument (GE Healthcare Life Sciences, Pittsburgh, PA, USA) at 25 °C. The binding responses of peptides at different concentrations were measured. PBS-P buffer containing 1% DMSO was filtered through a 0.22 μm membrane filter and degassed before use. Recombinant human PD-L1 was diluted to 10 μg/mL with sodium acetate solution at pH 4.5 and covalently coated on a CM5 sensor chip by using amine-coupling kit (BR-1000-50, GE Healthcare Life Sciences, Pittsburgh, PA, USA). The PD-L1 protein (25 kDa, Abcam, Cambridge, UK) was immobilized on the chip to about 5600 RU level of response. The blank channel was used as a negative control for background correction in each assay. The rest of the binding sites on the sensor chip were blocked by ethanolamine. Peptide C5 was dissolved in DMSO and diluted with PBS-P solution to desired concentrations. Peptide S2 was dissolved with PBS-P solution (20 mM phosphate buffer, 0.05% surfactant P20 pH = 7.4, 137 mM NaCl, and 2.7 mM KCl) to desired concentrations. Each sample was injected onto the sensor chip at a flow rate of 30 µL/min for 60 s (contact phase), followed by 60 s of buffer flow (dissociation phase).

## 4. Conclusions

To our knowledge, PD-L1 ascidian peptide inhibitors have not been reported yet. In this study, potential PD-L1 peptide inhibitors were screened in ascidian enzymatic hydrolysates using the AUF-nanoLC-MS/MS method, which was shown to be a highly efficient method. The 50 mg/mL of enzymatic hydrolysates of the ascidians *Ciona intestinalis* and *Styela clava* exhibited an inhibition ratio of 96.6% and 99.2% against PD-1/PD-L1 interaction, respectively, indicating that the ascidian enzymatic hydrolysates were bioactive and contained PD-1/PD-L1 inhibitory components.

Using the AUF-nanoLC-MS/MS method, two PD-L1 peptide inhibitors (C5 and S2) were obtained from *Ciona intestinalis* and *Styela clava* hydrolysates, respectively, which showed a concentration-dependent inhibition on PD-1/PD-L1 interaction. The IC_50_ values of C5 and S2 were 33.9 μM and 112.8 μM, respectively. The K_D_ values of C5 and S2 were 22.9 µM and 29.1 µM, respectively. C5 and S2 exhibited moderate effects against PD-1/PD-L1 interaction, suggesting their potential immunotherapeutic value. The source of C5 (LDVVIHTVTYGDR) was supposed to be possibly from *Streptomyces* which are the dominant genus in many ascidians. The sequence of S2 (VLRDNIQGITKPAIR) matches exactly the fragment of Histone H4 of *Ciona savignyi*.

It should be noted that the water-solubility of C5 is poor and that of S2 is good. Therefore, it may be necessary to increase the water solubility of C5 and improve its bioavailability by structural modifications in future studies.

## Figures and Tables

**Table 1:** Candidate PD-L1 peptide binders from *Ciona intestinalis* hydrolysates by the AUF-nanoLC-MS/MS method.

ID	Sequence	Candidate Protein Name (Organism)	Mass (Da)
**C1**	TTGIVFDSGDGVSHTVPIYEGYALPHAILR	Actin, and CsCA1 (Transparent ascidian)	3186.55
C2	KDLYANTVLSGGSTMFPGIADR	Actin (Transparent ascidian)	2313.57
C3	TVTFEEFLPMLAQIK	EF-hand domain-containing protein (Transparent ascidian)	1767.10
**C4**	AGFAGDDAPRAVFPSIVGRPR	Actin (Rotaria magnacalcarata, Rotifer, Caenorhabditis latens), Beta-actin (Nematostella vectensis (Starlet sea anemone)), and ATP-binding cassette sub-family B member 10 (Asian catfish)	2156.39
**C5**	LDVVIHTVTYGDR	Uncharacterized protein (Streptomyces albireticuli, Streptomyces sp. ISL-11, Streptomyces tubercidicus) ^a^	1487.67
**C6**	QYEDMFGEDLVDR	Annexin (Transparent ascidian)	1616.70
**C7**	QVNMANHLSKDSR	RING-type E3 ubiquitin transferase (Transparent ascidian)	1499.65
**C8**	PLAIDLLHPSPEEEK	40S ribosomal protein S27 (Transparent ascidian)	1687.88
C9	ISVDVNIPADAIIGR	Transglutaminase-like domain-containing protein (Transparent ascidian) ^a^	1552.79
C10	PGAGGKSSTYGR	Transcription elongation factor SPT5 (Transparent ascidian)	1137.20
C11	LLDPEDIVVERPDEK	Spectrin beta chain (Transparent ascidian)	1766.94
C12	FVDHIMDDQVVEDLTIK	Dolichyl-diphosphooligosaccharide-protein glycosyltransferase subunit 1 (Transparent ascidian)	2017.27
C13	HLDIPQMLDAQELVDMAKPDER	Calponin-homology (CH) domain-containing protein, and Actinin, alpha 1 (Transparent ascidian)	2564.87
C14	DYEEAVMQNDATVGQLK	Myosin motor domain-containing protein (Transparent ascidian)	1911.05
C15	SNAVGITWSPVEGAEK	Uncharacterized LOC100184933 (Transparent ascidian)	1644.79
C16	VSYEESIEQLEIVK	Myosin motor domain-containing protein (Transparent ascidian)	1665.85
C17	NLLAVAAETDITFPEAEK	60S acidic ribosomal protein P0, and Large ribosomal subunit protein uL10 (Transparent ascidian)	1932.12
C18	IGTSGGLGLDPGTVVITDK	Nucleoside phosphorylase domain-containing protein (Transparent ascidian)	1800.03
C19	NDLTLQLQAEQDNLADAEER	Myosin motor domain-containing protein (Transparent ascidian) ^a^	2286.35
C20	TIAMDSTER	ATP synthase subunit beta (Transparent ascidian)	1023.11
C21	TLHPEVDEDLIER	Obscurin (Transparent ascidian)	1565.68
C22	HGIVEDWDLMEK	Actin-related protein 3 (Transparent ascidian)	1471.64
C23	EAIANVQDQIADLDK	Large ribosomal subunit protein uL13 (Transparent ascidian)	1642.77
C24	LEMQEIQLTEAK	Tropomyosin (Transparent ascidian) ^a^	1432.64
C25	LEHINHEK	Eukaryotic translation initiation factor 3 subunit A (Transparent ascidian)	1019.13
C26	EILIEDEGELKDIYETFPIDLK	NTR domain-containing protein (Transparent ascidian)	2622.92
C27	DVASALGDLINATK	FERM domain-containing protein (Transparent ascidian)	1387.52
C28	GINLPGIEVDLPAVSEK	Pyruvate kinase (Transparent ascidian)	1750.99
C29	LHEEEIEDLKEQIK	IF rod domain-containing protein (Transparent ascidian) ^a^	1752.93
C30	TTNVVVADLSESK	Alcohol dehydrogenase-like N-terminal domain-containing protein (Transparent ascidian)	1362.48
C31	KKDEEEIEELR	Troponin T (Transparent ascidian)	1417.52
C32	AGVLADLEDK	Myosin type-2 heavy chain 2 (Penicillium cataractarum) ^a^, Myosin motor domain-containing protein, Myosin tail, and Myosin type II heavy chain (Penicillium) ^a^	1030.12
C33	EILQGESNVQEVK	Serine/threonine-protein phosphatase (Transparent ascidian)	1472.62
C34	IEDLSGGELQR	ATP-binding cassette sub-family E member 1 (Transparent ascidian)	1216.30
C35	EILIEDEGELK	NTR domain-containing protein (Transparent ascidian)	1287.42
C36	SELDDELGR	IF rod domain-containing protein (Transparent ascidian)	1033.03
C37	DGFIDKEDLK	EF-hand domain-containing protein, and Myosin regulatory light chain (Transparent ascidian)	1179.28
C38	TSLEEQLEEEEESR	Myosin-10, Myosin tail domain-containing protein (Transparent ascidian)	1707.69
C39	IVPPEDGDDEK	RRM domain-containing protein (Transparent ascidian)	1213.25
C40	EGIFEEEIK	Tropomyosin (Transparent ascidian)	1093.20
C41	DAEEIEKDEQVAAEK	Small ribosomal subunit protein uS2 (Transparent ascidian)	1703.76
C42	AEQAEADKK	Beta-lactamase-related domain-containing protein (Patagioenas fasciata monilis), and DAK2 domain fusion protein YloV (Lachnospiraceae bacterium M18-1)	989.03

^a^ Incomplete match. Bold sequences indicate the peptides to be synthesized.

**Table 2:** Energy scores, ranker scores, and isoelectric points of Candidate PD-L1 peptide binders from *Ciona intestinalis* hydrolysates by the AUF-nanoLC-MS/MS method ^a^.

ID	Sequence	Energy Score	Ranker Score	Isoelectric Point
**C1**	TTGIVFDSGDGVSHTVPIYEGYALPHAILR	−213.328	0.136	5.07
C2	KDLYANTVLSGGSTMFPGIADR	−205.126	0.049	6.56
C3	TVTFEEFLPMLAQIK	−199.778	0.218	4.15
**C4**	AGFAGDDAPRAVFPSIVGRPR	−195.369	0.532	10.65
**C5**	LDVVIHTVTYGDR	−187.135	0.118	5.04
**C6**	QYEDMFGEDLVDR	−185.745	0.092	3.25
**C7**	QVNMANHLSKDSR	−183.074	0.184	9.83
**C8**	PLAIDLLHPSPEEEK	−179.346	0.17	4.04
C9	ISVDVNIPADAIIGR	−175.185	0.386	3.71
C10	PGAGGKSSTYGR	−173.527	0.343	10.71
C11	LLDPEDIVVERPDEK	−172.11	0.401	3.61
C12	FVDHIMDDQVVEDLTIK	−170.133	0.097	3.61
C13	HLDIPQMLDAQELVDMAKPDER	−169.437	0.123	3.85
C14	DYEEAVMQNDATVGQLK	−168.407	0.412	3.43
C15	SNAVGITWSPVEGAEK	−167.176	0.08	4.15
C16	VSYEESIEQLEIVK	−166.506	0.202	3.67
C17	NLLAVAAETDITFPEAEK	−161.999	0.28	3.54
C18	IGTSGGLGLDPGTVVITDK	−161.344	0.072	3.71
C19	NDLTLQLQAEQDNLADAEER	−160.497	0.209	3.19
C20	TIAMDSTER	−158.804	0.083	3.93
C21	TLHPEVDEDLIER	−158.752	0.082	3.79
C22	HGIVEDWDLMEK	−158.583	0.11	3.93
C23	EAIANVQDQIADLDK	−155.919	0.126	3.32
C24	LEMQEIQLTEAK	−155.906	0.083	3.85
C25	LEHINHEK	−153.529	0.17	6.06
C26	EILIEDEGELKDIYETFPIDLK	−152.172	0.068	3.48
C27	DVASALGDLINATK	−149.681	0.088	3.71
C28	GINLPGIEVDLPAVSEK	−146.833	0.088	3.69
C29	LHEEEIEDLKEQIK	−144.492	0.298	4.08
C30	TTNVVVADLSESK	−142.701	0.079	3.93
C31	KKDEEEIEELR	−142.135	0.133	4.08
C32	AGVLADLEDK	−141.924	0.094	3.54
C33	EILQGESNVQEVK	−141.694	0.136	3.85
C34	IEDLSGGELQR	−140.846	0.056	3.69
C35	EILIEDEGELK	−135.713	0.119	3.44
C36	SELDDELGR	−134.834	0.095	3.43
C37	DGFIDKEDLK	−133.987	0.303	3.82
C38	TSLEEQLEEEEESR	−132.64	0.249	3.37
C39	IVPPEDGDDEK	−131.107	0.032	3.25
C40	EGIFEEEIK	−129.198	0.139	3.67
C41	DAEEIEKDEQVAAEK	−124.001	0.112	3.61
C42	AEQAEADKK	−119.713	0.067	4.32

^a^ Energy scores of peptides binding with PD-L1 (PDB code: 5J89) in kcal/mol calculated by HPEPDOCK. Ranker scores and isoelectric point values were predicted by Peptide Ranker. Bold sequences indicate the peptides to be synthesized.

**Table 3:** Candidate PD-L1 peptide binders from the *Styela clava* hydrolysates by AUF-nanoLC-MS/MS method.

ID	Sequence	Candidate Protein Name (Organism)	Mass
**S1**	FLEIFTQR	Annexin (Transparent ascidian)	1053.22
**S2**	VLRDNIQGITKPAIR	Histone H4 (Transparent ascidian)	1694.01
**S3**	AFILPEGVSAER	Body wall muscle protein HR-29 (Sea squirt)	1288.44
S4	VWLDPNETSMISNANSR	Ribosomal protein L19 (Transparent ascidian) ^a^	1934.08
S5	VDTLMVRNNLR	Tetratricopeptide repeat protein (Streptomyces sp. Rer75) ^a^, and Oxidoreductase (Actinomadura sp. WAC 06369) ^a^	1330.55
S6	VAPEEHPVLLTEAPLNPKANR	Beta-actin, and Alpha-actin (Rotaria magnacalcarata), Actin (Rotaria socialis)	2295.58
S7	AVMSLQMEMQQIMK	Glutamine--fructose-6-phosphate transaminase (Transparent ascidian) ^a^	1668.08
S8	GYEEWLISEMR	Actinin, alpha 1, Calponin-homology (CH) domain-containing protein, and EF-hand domain-containing protein (Transparent ascidian) ^a^	1412.57
S9	TVQTLNLEIDSMR	IF rod domain-containing protein, and Glial fibrillary acidic protein (Transparent ascidian) ^a^	1519.72
S10	VLGSGTNLDSAR	L-lactate dehydrogenase (Transparent ascidian)	1189.26
S11	FTGMLSMLDDPEPFAR	Body wall muscle protein HR-29 (Sea squirt)	1827.06
S12	EELDMEHRRSK	Uncharacterized protein (Transparent ascidian)	1429.55
S13	DGILQIDAPVAVAIDNK	Body wall muscle protein HR-29 (Sea squirt)	1751.98
S14	MTEQWMK	Carboxylic ester hydrolase (Transparent ascidian)	953.14
S15	LVMVEAELERGEER	Tropomyosin (Transparent ascidian)	1659.85
S16	LLEAQIATGGLIDPR	Uncharacterized protein (Transparent ascidian)	1566.80
S17	KLETLQEELELLK	IF rod domain-containing protein, and Glial fibrillary acidic protein (Transparent ascidian) ^a^	1585.83
S18	GVDLDQLLDMSR	Small ribosomal subunit protein uS19 (Transparent ascidian)	1361.50
S19	ANAEVANWR	Myosin motor domain-containing protein (Transparent ascidian)	1030.09
S20	MAGTSDCVKR	Uncharacterized LOC100177244 (Transparent ascidian)	1067.24
S21	LSGGDIESYLLEK	Myosin motor domain-containing protein (Transparent ascidian)	1423.56
S22	NIKEGDIVKR	ATP synthase subunit alpha (Transparent ascidian)	1171.37
S23	VVSQTEDVR	Glial fibrillary acidic protein (Transparent ascidian)	1032.11
S24	EDDVQQMNPPK	Myosin motor domain-containing protein (Transparent ascidian) ^a^	1300.40

^a^ Incomplete match. Bold sequences indicate the peptides to be synthesized.

**Table 4:** Energy scores, ranker scores and isoelectric points of Candidate PD-L1 peptide binders from the *Styela clava* hydrolysates by AUF-nanoLC-MS/MS method ^a^.

ID	Sequence	Energy Score	Ranker Score	Isoelectric Point
**S1**	FLEIFTQR	−191.311	0.335	6.62
**S2**	VLRDNIQGITKPAIR	−182.923	0.15	11.23
**S3**	AFILPEGVSAER	−178.808	0.255	4.15
S4	VWLDPNETSMISNANSR	−175.773	0.248	3.93
S5	VDTLMVRNNLR	−175.323	0.154	10.65
S6	VAPEEHPVLLTEAPLNPKANR	−175.164	0.221	5.36
S7	AVMSLQMEMQQIMK	−174.227	0.215	6.91
S8	GYEEWLISEMR	−172.518	0.575	3.85
S9	TVQTLNLEIDSMR	−170.263	0.09	3.93
S10	VLGSGTNLDSAR	−169.041	0.185	6.61
S11	FTGMLSMLDDPEPFAR	−167.011	0.316	3.54
S12	EELDMEHRRSK	−165.086	0.128	5.39
S13	DGILQIDAPVAVAIDNK	−164.379	0.282	3.41
S14	MTEQWMK	−160.485	0.349	6.61
S15	LVMVEAELERGEER	−158.101	0.061	3.97
S16	LLEAQIATGGLIDPR	−157.057	0.246	3.93
S17	KLETLQEELELLK	−156.352	0.118	4.15
S18	GVDLDQLLDMSR	−155.986	0.374	3.41
S19	ANAEVANWR	−155.121	0.413	6.93
S20	MAGTSDCVKR	−153.381	0.307	8.6
S21	LSGGDIESYLLEK	−149.843	0.315	3.69
S22	NIKEGDIVKR	−149.659	0.127	9.53
S23	VVSQTEDVR	−144.2	0.063	3.93
S24	EDDVQQMNPPK	−134.958	0.22	3.54

^a^ Energy scores of peptides binding with PD-L1 (PDB code: 5J89) in kcal/mol calculated by HPEPDOCK. Ranker scores and isoelectric point values were predicted by Peptide Ranker. Bold sequences indicate the peptides to be synthesized.

**Table 5:** Interactions between C5 or S2 and dimeric PD-L1 ^a^.

Ligand		PD-L1 ^b^		Interaction	Distance (Å)	E (kcal/mol)
	His6	OE1	_A_Glu60	H-donor	2.83	−2.3
	Thr7	OE1	_A_Glu60	H-donor	2.80	−3.5
	Tyr10	O	_A_Glu60	H-donor	2.90	−2.3
	Arg13	OE1	_A_Glu60	H-donor	3.24	−2.6
	Arg13	O	_A_Glu60	H-donor	2.72	−3.1
	Asp2	NZ	_B_Lys129	H-acceptor	2.62	−12.7
C5	Asp12	OG1	_B_Thr127	H-acceptor	2.75	−3.8
	Asp12	NE	_B_Arg125	H-acceptor	2.79	−4.8
	Asp12	NH2	_B_Arg125	H-acceptor	3.12	−2.2
	Asp12	NH2	_B_Arg125	H-acceptor	2.95	−1.6
	Asp2	NZ	_B_Lys129	ionic	2.62	−7.6
	Asp12	NE	_B_Arg125	ionic	2.79	−6.0
	Asp12	NH2	_B_Arg125	ionic	3.12	−3.7
	Arg13	OE1	_A_Glu60	ionic	3.24	−3.1
	Val1	OD2	_A_Asp26	H-donor	2.78	−14.1
	Arg15	O	_A_Arg125	H-donor	2.82	−4.8
	Arg15	OD2	_A_Asp26	H-donor	2.75	−4.6
	Asp4	OG1	_A_Thr127	H-acceptor	2.61	−3.5
	Gln7	NZ	_A_Lys129	H-acceptor	2.78	−11.5
	Thr10	NE2	_B_His78	H-acceptor	2.88	−3.0
	Arg15	N	_A_Arg125	H-acceptor	3.43	−1.5
S2	Arg15	NH2	_A_Arg125	H-acceptor	2.67	−3.9
	Val1	OD1	_A_Asp26	ionic	2.69	−7.0
	Val1	OD2	_A_Asp26	ionic	2.78	−6.1
	Arg15	OD2	_A_Asp26	ionic	3.75	−1.1
	Arg15	OD2	_A_Asp26	ionic	2.75	−6.4
	Arg15	NE	_A_Arg125	ionic	3.93	−0.6
	Arg15	NH2	_A_Arg125	ionic	3.89	−0.7
	Arg15	NE	_A_Arg125	ionic	3.43	−2.2
	Arg15	NH2	_A_Arg125	ionic	2.67	−7.1

^a^ Energy decompositions calculated by MOE using the Amber 10 force field. ^b^ PD-L1 (PDB: 5J89).

## Data Availability

The data presented in this study are available on request from the corresponding author.

## Supplementary Materials

The following supporting information can be downloaded at: https://www.mdpi.com/article/10.3390/md23040137/s1, Figure S1: HPLC and MS spectra of 9 synthesized ascidian peptides.
