# Supplementary material for: Discovery of PD-L1 Peptide Inhibitors from Ascidian Enzymatic Hydrolysates by Affinity Ultrafiltration Coupled to NanoLC-MS/MS
Source: Mar Drugs. 2025 Mar 21;23(4):137. doi: 10.3390/md23040137 (PMC12028738; doi:10.3390/md23040137)
Supplement: Supplementary file 1 [file marinedrugs-23-00137-s001.zip › marinedrugs-3488524-supplementary.pdf]

Supplementary Materials

Discovery of PD-L1 Peptide Inhibitors from Ascidian Enzymatic Hydrolysates by Affinity Ultrafiltration Coupled to NanoLC-MS/MS

Qiuyang Huang [1], Xiaoling Zang [1, 2, *], Xinyu Jin [1], Qian Liu [1], Xin Zhang [1, 3], Xinyu Li [1], Lizhen Zhao [4], Zhihua Lv [1, 2, *]

[1] School of Medicine and Pharmacy, Ocean University of China, Qingdao, 266003, China

[2] Laboratory for Marine Drugs and Bioproducts, Qingdao Marine Science and Technology Center, Qingdao, 266237, China

[3] Sinopep Allsino Bio Pharmaceutical Co.,Ltd., Hangzhou, 310020, China

[4] College of Physics, Qingdao University, Qingdao, 266071, China

*Corresponding authors: Xiaoling Zang, zangxiaoling@ouc.edu.cn;
Zhihua Lv, lvzhihua@ouc.edu.cn

Table of contents

Figure S1. HPLC and MS spectra of 9 synthesized ascidian peptides.

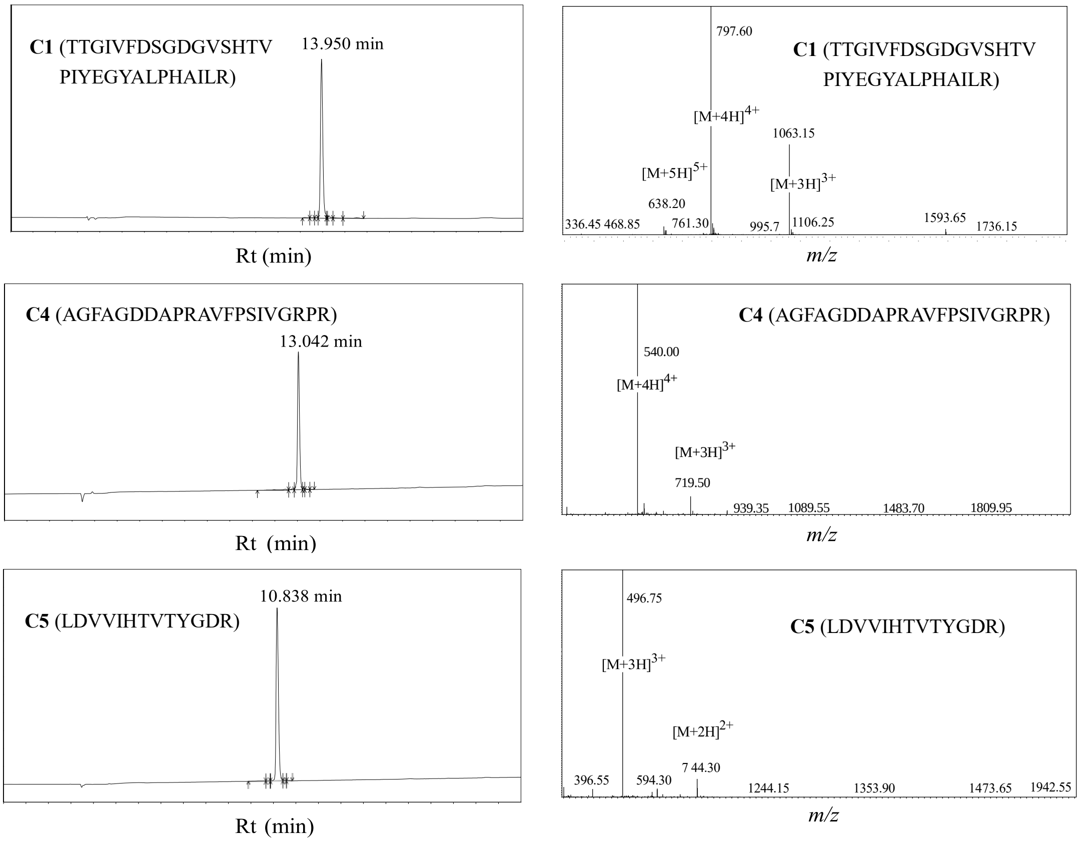

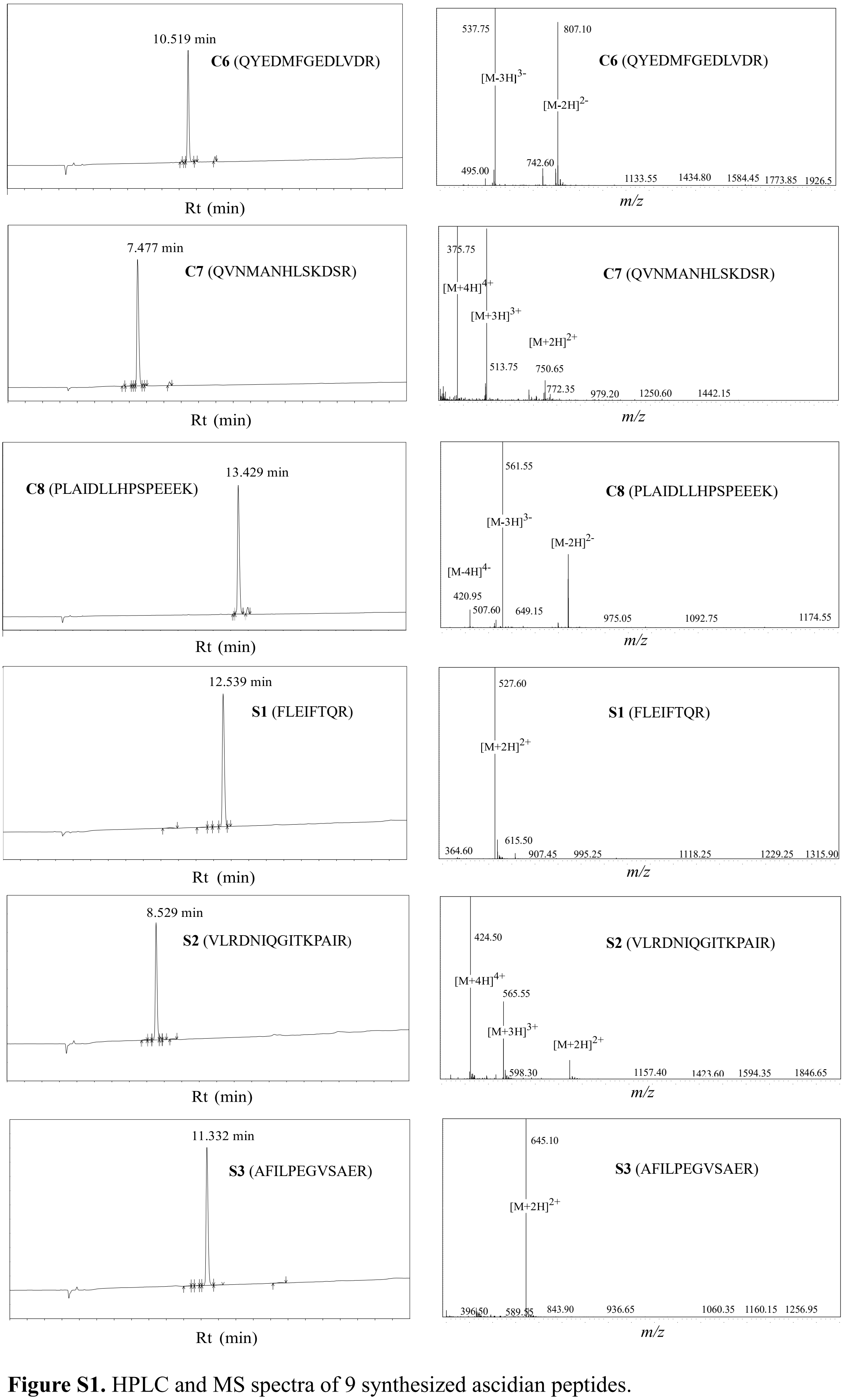

Figure S1. HPLC and MS spectra of 9 synthesized ascidian peptides.
